# Effect of Dietary Fiber and Thermal Conditions on Rice Bran Wax-Based Structured Edible Oils

**DOI:** 10.3390/foods10123072

**Published:** 2021-12-10

**Authors:** Laura Principato, Daniele Carullo, Andrea Bassani, Alice Gruppi, Guillermo Duserm Garrido, Roberta Dordoni, Giorgia Spigno

**Affiliations:** Department of Sustainable Food Product (DiSTAS), Università Cattolica del Sacro Cuore, Via Emilia Parmense 84, 29122 Piacenza, Italy; laura.principato@unicatt.it (L.P.); daniele.carullo@unicatt.it (D.C.); alice.gruppi@unicatt.it (A.G.); guillermo.dusermgarrido@unicatt.it (G.D.G.); roberta.dordoni@unicatt.it (R.D.); giorgia.spigno@unicatt.it (G.S.)

**Keywords:** food rheology, oleogelation, mathematical models, texture, edible oil structuring

## Abstract

In this work, extra-virgin olive oil (EVO)- and sunflower oil (SFO)-based oleogels were structured using rice bran wax (RBW) at 10% by weight (*w*/*w*). Bamboo fiber milled with 40 (BF_40_), 90 (BF_90_) and 150 (BF_150_) µm of average size was added as a structuring agent. The effect of fiber addition and cooling temperature (0, 4, and 25 °C) on thermal and structural parameters of achieved gels was assessed by rheological (both in rotational and oscillatory mode), texture, and differential scanning calorimetry tests. Oleogelation modified the rheological behavior of EVO and SFO, thus shifting from a Newtonian trend typical of oils to a pseudoplastic non-Newtonian behavior in gels. Moreover, oleogels behaved as solid-like systems with G′ > G″, regardless of the applied condition. All samples exhibit a thermal-reversible behavior, even though the presence of hysteresis suggests a partial reduction in structural properties under stress. Decreasing in cooling temperature negatively contributed to network formation, despite being partially recovered by low-granulometry fiber addition. The latter dramatically improved either textural, rheological, or stability parameters of gels, as compared with only edible oil-based systems. Finally, wax/gel compatibility affected the crystallization enthalpy and final product stability (gel strength) due to different gelator–gelator and gelator–solvent interactions.

## 1. Introduction

The food industry is continuously facing new challenges related to innovative ingredients and final product development. The request for change in formulation is driven by both nutritional and sensorial consumers’ expectations. Nowadays, finding fulfilling fat alternatives is one of the most difficult goals to achieve. In particular, the focus is centered on the substitution of saturated fats (especially of animal origin) with possible solutions that guarantee similar sensory and structural properties by enhancing the nutritional profile and lowering caloric intake [1,2]. Furthermore, despite palm oil being widely used due to its technological suitability, it is characterized by a low nutritional value (saturated fatty acids content) and environmental issues [3].

The current routes to fat substitution are based on the exploitation of carbohydrates (cellulose, starches, gums, maltodextrins, fibers), proteins (milk, whey, egg yolk), or modified lipid systems [4]. Nevertheless, carbohydrate- and protein-based solutions present some limitations such as poor compatibility with oil phase or stability issues and are used for partial replacement only [4].

Instead, modified lipid systems are solidified liquid oils that are highly similar to saturated fats. However, the currently available commercial solutions are produced by chemical reaction (hydrogenation) and/or enzymatic treatments (inter-esterification) that increase the saturated fraction, thus affecting their nutritional value and, in some cases, producing some negative side compounds as trans fatty acids have a harmful effect for human health [5,6].

More recently, wide attention has been paid to oleogelation as an advanced and alternative route of structuring [4]. Oleogel systems might be differentiated based on gelator type and route of gel formation mechanism. Specifically, gels can be obtained by direct or indirect methodology. Direct oleogels are mainly lipid-based systems in which liquid oils are transformed in the gel form without the occurrence of chemical/enzymatic reactions [7]. The physical transition takes place due to the addition of gelling agents capable of rearranging themselves in 3D networks entrapping the liquid phase [7]. The great potential of oleogelated systems is related to a full substitution of solid fats with gels containing unsaturated or poly-unsaturated edible oil in their native form [5]. Besides, the gelling agents not only enable preserving the nutritional characteristics of the oil but could even preserve them from oxidation phenomena to which unsaturated bonds are prone to be subjected [8,9].

There is a broad variety of direct gelling agents that operate, alone or in combination, throughout different mechanisms of gelation. In particular, natural waxes are among the most exploited ones, such as those belonging to sunflower, carnauba, candelilla, and rice bran [10,11,12,13]. Such materials are widely used for their gelling capacity, as well as for their capability to form thermo-reversible gels [14]. Moreover, they are economical and easy to use by direct dispersion in the liquid phase. Among organogelators used in direct dispersion, rice bran wax (RBW) has proven to be one of the most promising novel ingredients due to its ability to structure oils at very low concentrations. Furthermore, RBW is an innovative and sustainable ingredient produced in large quantities as a by-product of rice bran oil production [9,15,16]. It is worth inferring that the gel strength is strictly related to either the gelation condition or the wax concentration. However, increasing the percentage of wax within the formulation could strongly affect product taste. Hence, the main challenge lies in improving the mechanical properties of lipid-based gel systems at reduced gelling agent concentration [17].

This work aimed to investigate the effect of fiber addition in the preparation of rice bran wax (RBW)-based oleogels for potential food applications. As a target, bamboo fiber was selected due to high insoluble fiber content enriched in lignocellulose derivative, thus promoting wide use in food fortification [18,19,20]. In this perspective, the effects of cooling condition and bamboo fiber addition on extra-virgin olive oil (EVO) and sunflower oil (SFO) oleogels structured using RBW at 10% by weight (*w*/*w*) were evaluated. Specifically, rheological (both in rotational and oscillatory mode), texture, and differential scanning calorimetry tests were carried out to assess thermal and structural parameters of achieved gels. Within this frame, dietary fibers are well known for their outstanding properties as thickening agents in water-based systems, as well as for their capability to possibly increase the nutritional profile of final products [21]. However, plant fibers have many free hydroxyl groups at the molecular level that easily bond with oil or water [22]. Therefore, they generally exhibit a good affinity for both oil and water, which may potentially justify their exploitation as potential stabilizing and structuring agents for oleogels systems.

Based on our knowledge, the use of cellulose as a gelling agent is widely known. However, cellulose is mostly utilized in microcrystalline particulate form to stabilize oil-in-water (o/w) Pickering emulsions [23,24,25,26], and only a few studies have reported cellulose used in an o/w emulsion-template approach [27,28]. Moreover, to date no research has involved the dispersion of dietary fiber directly in the oil phase, including in a direct oleogelation method.

## 2. Materials and Methods

### 2.1. Raw Materials

Extra-virgin olive oil (EVO) and refined not-high oleic sunflower oil (SFO) used in this research work were purchased from a local supermarket and stored in dark conditions at room temperature (T = 25 °C) until use. Rice bran wax (RBW) was purchased from Strahl & Pitsch, Inc. (West Babylon, NY, USA), whereas bamboo fibers of different granulometry (40, 90, and 150 µm), namely BF**_40_**, BF**_90_**, and BF**_150_**, were kindly supplied by Prodotti Gianni S.r.l. (Milan, Italy).

### 2.2. Bamboo Fiber Characterization

#### 2.2.1. Total, Soluble, and Insoluble Dietary Fiber Content

Total dietary fibers and their fractions (soluble and insoluble) of all the investigated samples were determined using a K-TDFR-200A Kit (Megazime, Wicklow, Ireland) based on the AOAC method 991.43. Soluble and insoluble dietary fibers were obtained as indigestible residues after enzymatic digestion of non-dietary fiber components. The analyses of residual ash and protein contents were carried out in the residues for the corresponding corrections.

#### 2.2.2. Water Holding Capacity (WHC)

For the analyses, 0.5 g of fiber were weighed in a Falcon tube, to which 10 mL of distilled water were added. Afterward, the mixture was vortexed for 90 s and incubated in a water heating bath (OBC, Velp Scientifica, Usmate Velate, Italy) at 60 °C for 30 min and then in cold water for the same time. Falcons were then centrifuged (SL 16R, Thermo Fisher Scientific, Waltham, MA, USA) at 25 °C for 20° min by setting a rotor speed of 3075 g. The obtained supernatant was discarded and the remaining solid pellet was weighed. WHC was determined as reported in Equation (1):

(1) $$WHC\;\left(\frac{g}{g}\right)=\frac{g_{final}-g_{initial}}{g_{initial}}\;$$

where g_final_ stands for the weight of pellet left after centrifugation, while g_initial_ represents the initial mass of sample (fiber).

#### 2.2.3. Oil Holding Capacity (OHC)

Similarly, oil holding capacity (OHC) was determined by weighing 1 g of fiber in a Falcon tube. Then, 10 mL of sunflower oil and extra-virgin olive oil were alternatively added, and the mixture was vortexed for 30 s. Falcon tubes were transferred in a centrifuge and processed at 25 °C for 10 min with a rotor speed of 3700× *g*. The supernatant was discarded and the remaining solid pellet was weighed. OHC was determined according to Equation (2):

(2) $$OHC\;\left(\frac{g}{g}\right)=\frac{g_{final}-g_{initial}}{g_{initial}}\;$$

where g_final_ stands for the weight of pellet left after centrifugation, while g_initial_ represents the initial mass of sample (fibers).

#### 2.2.4. Particle Size Distribution (PSD)

PSD measurements were executed via a Mastersizer 3000 (Malvern Instruments, Worcestershire, UK) in a dimension range varying from 0.01 to 10,000 µm. The instrument was equipped with an AERO S system for solid particle determination and the measurement parameters were set as follows: 10% of laser obscuration, 1.52 and 1.33 of refractive index for particles and background, respectively. Measurements were carried out in triplicate. The results were analyzed via Malvern software and expressed either in volume percentage (%) or mean diameter (D_4,3_; µm).

### 2.3. Preparation of the Oleogels

**Figure 1:**
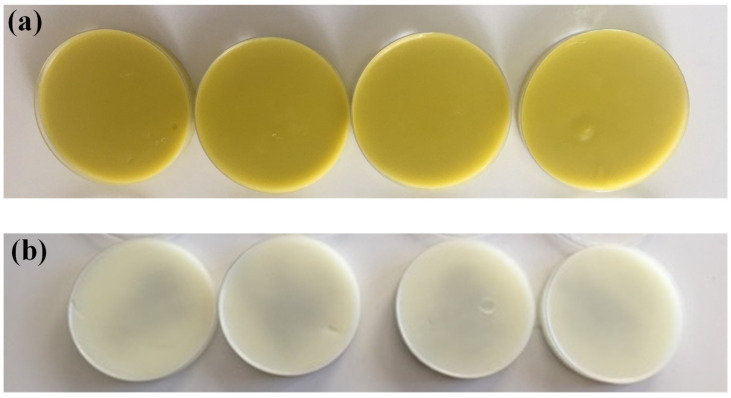
Pictures of oleogels prepared from extra-virgin olive oil (**a**) and sunflower oil (**b**), using rice bran wax as a gelling and structuring agent at a concentration of 10% (*w*/*w*).

To form the oleogels (Figure 1), weighed proportions of EVO/SFO oil and RBW (90–10% *w*/*w*) and, eventually, bamboo fibers (0.5% wt/wt), were mixed in shearing condition and heated up in a water bath to 85 °C until complete dissolution. Then, the liquid mixture was gently poured into Petri dishes and cooled down for 1 h in three different ways, including at 0 °C in an ice bowl, 4 °C in the fridge, and 25 °C at room temperature, before being stored overnight for further analyses.

### 2.4. Texture Analysis

Texture analysis of oleogels was performed in penetration mode using a Texture Analyzer (TVT 6700, Perten, Sweden), equipped with a 5 mm cylindrical probe. Briefly, 20 g of sample were poured into a Petri dish (7.5 cm diameter) until reaching an initial height of 1 cm to avoid loss of product and then the probe was used to penetrate the sample up to 50% of its initial volume. The test speed was set at 1 mm/s. Gel strength, hardness, work of penetration, as well as stickiness and adhesiveness attributes were computed by instrument software from the obtained force-time curves. Specifically, gel strength was measured at 25% of volume penetration, hardness was defined as the force implied during breakage, and work of penetration was obtained as the area standing below the compression peak. Conversely, stickiness and adhesiveness were defined as maximum force and area of negative peak obtained during probe retraction, respectively.

### 2.5. Differential Scanning Calorimetry Analysis

A differential scanning calorimeter (microDSC Setaram, Caluire, France) was used to reveal the thermal behavior of achieved oleogels during cooling/heating cycles. For the sake of analysis, 0.8 g of each sample was weighed and placed in Hastelloy capsules. An empty capsule of equal weight (6.531 ± 0.005 g) was used as a reference. The scanning temperature was raised from 5 °C to 100 °C at a rate of 0.7 °C/min. The latter temperature was maintained for 15 min, then cooled to 25 °C at a rate of 1 °C/min. As far as the melting/crystallization phenomena are concerned, the onset (Tonset) and the offset (Toffset) temperatures are defined as the intersection of the tangents of the characteristic peak with the extrapolated baseline, and the peak temperature is defined as the temperature at the maximum/minimum of the corresponding thermal event (or peak). The area under the main detected peak represents the energy (J/g) involved during the interesting phenomenon.

### 2.6. Rheological Measurements

Rotational and oscillatory measurements were carried out using a controlled-stress MCR-302 rheometer (Anton Paar, Gratz, Austria) provided with rough plates geometry. The plates were 25 mm in diameter and a 1 mm gap was selected to perform the tests. The rheological behavior of gels was analyzed in either static (rotational) or dynamic (oscillatory) conditions.

#### 2.6.1. Rotational Rheology

Steady-state flow tests were carried out for all the samples at a temperature of 25 °C since gels were developed with the aim to be shelf-stable (room temperature). The shear rate ($\dot{\gamma }$) was set between 0.01 s^−1^ and 100 s^−1^, with the flow curves (*η* vs. $\dot{\gamma }$) being analyzed using the Power Law model [29] to describe the rheological behavior of achieved oleogels.

#### 2.6.2. Oscillatory Rheology

The amplitude strain sweep test was assessed to determine the linear viscoelastic region (LVR) for the investigated samples. To this purpose, the frequency value was kept constant (1 Hz) while the amplitude was allowed to vary within the range of 0.01–100%. Instead, for frequency sweep tests performed at a constant shear strain of 0.02%, values from 100 to 0.1 Hz were considered. The viscoelastic parameters, including the storage modulus (*G*′), loss modulus (*G*″), and loss tangent (tan δ), were plotted as a function of the frequency.

#### 2.6.3. Temperature Sweep Rheology

The rheological behavior of the sol-gel transition of the oleogel was measured by performing a temperature sweep in both rotational and oscillatory mode according to the method reported by Farahnaky et al. [30]. In particular, the gel sample was loaded onto the pre-set Peltier plate (25 °C) of the rheometer and allowed to equilibrate for 5 min. Temperature dependence of viscosity, storage (*G*′), and loss (*G*″) moduli as well as complex modulus (G*) were measured by heating the systems from 25 to 100 °C. The temperature gradient was 2 °C/min on the heating scan. For rotational rheology, tests were carried out at a fixed shear rate of 50 s^−1^, while oscillatory measurements were performed at 0.2% of strain, which was well within the linear viscoelastic region, and 1 Hz of frequency. Before temperature sweep, dynamic torque sweeps were conducted to choose a common linear viscoelastic region for all samples.

### 2.7. Statistical Analysis

All analyses were repeated three times unless otherwise specified. The mean values and standard deviations (SD) of the experimental data were calculated. The influence of different processing parameters on structural and thermal properties of gels was evaluated using a one-way analysis of variance (ANOVA) followed by Tukey’s post-hoc test for means discrimination (*p* ≤ 0.05). The statistical analysis was executed via the IBM SPSS Statistics 21 software (SPSS Inc., Chicago, IL, USA).

## 3. Results and Discussions

### 3.1. Fiber Characterization

The main characteristic parameters of bamboo fibers employed in this study were schematized in Table 1. All samples showed the same ratio between soluble (SDFs) and insoluble (IDFs) dietary fibers. Specifically, IDFs and SDFs reached values equal to 92.32% and 1.92%, respectively, thus indicating a greater portion of cellulose, hemicelluloses, resistant starch, and lignin [31]. As can be seen from Table 1, similar capabilities of fibers in retaining water were recorded, irrespective of the sample average dimension. Conversely, samples characterized by higher granulometry (BF_90_, and BF_150_) showed a greater affinity towards lipids, as witnessed by the significantly greater (*p* < 0.05) values of either SOHC or OOHC values in comparison with those displayed by BF_40_ (Table 1).

**Figure 2:**
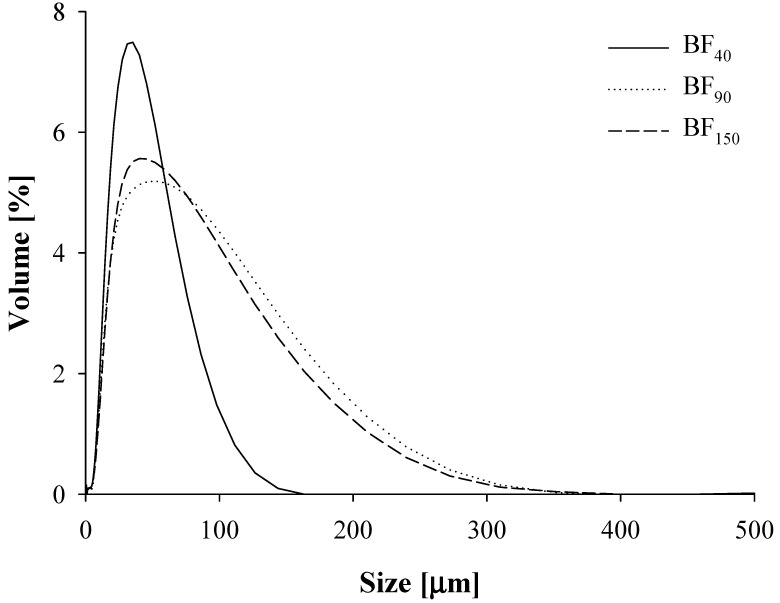
Particle size distribution (PSD) of the averaged curves, obtained by light scattering measurements, for the different bamboo fibers investigated in this study: bamboo fiber of 40 µm (BF_40_), bamboo fiber of 90 µm (BF_90_), and bamboo fiber of 150 µm (BF_150_).

Additional information on the size distribution of the fiber particulate was obtained from PSD measurements and results are depicted in the volume intensity curves of Figure 2. In particular, all samples exhibited a unimodal PSD, being characterized by a single peak which, depending on the analyzed fiber, ranged across a quite broad size span (0.01–300 µm). Plus, clear discrepancies can be denoted between the BF_40_ sample, which possesses a very sharp and narrow distribution around the mean value placed slightly below 50 µm, and bigger fibers. As a matter of fact, the latter group exhibited a less symmetrical shape and a wider distribution, thus suggesting the presence of larger particles, in full agreement with the detected greater D4.3 values (Table 1). However, significant differences between BF_90_ and BF_150_ were neither highlighted in terms of PSD curve shape nor regarding the D_4.3_ values (*p* > 0.05), conversely to what was declared by the producer.

### 3.2. Texture Analysis

**Figure 3:**
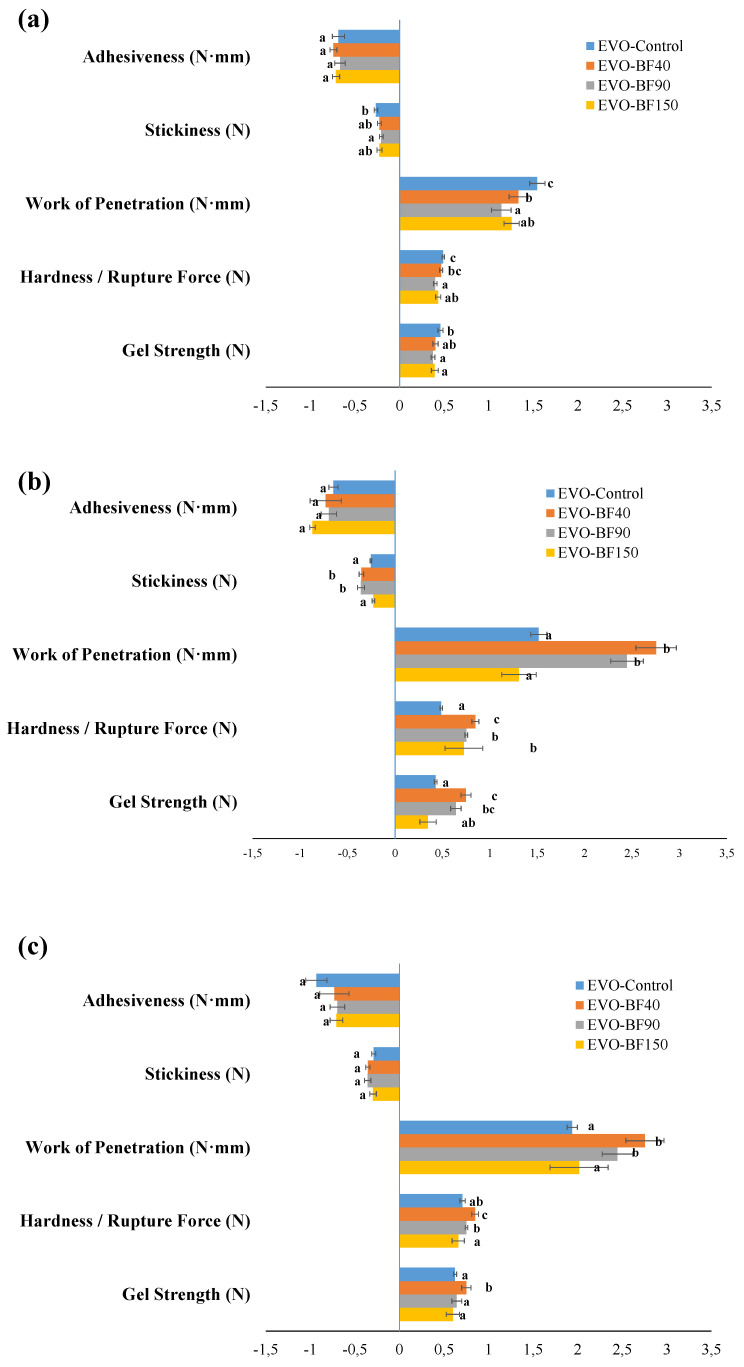
Values of gel strength (N), hardness, (N), work of penetration (N∙mm), stickiness (N), and adhesiveness (N∙mm) for the extra-virgin olive oil-based gels prepared at 25 °C (**a**), 4 °C (**b**), and 0 °C (**c**), as a function of the bamboo fiber average dimension. The samples were tested in a penetration mode with a cylindrical probe at 25 °C. The following systems were investigated: extra-virgin olive oil structured with 10% of rice bran wax (EVO-Control), extra-virgin olive oil structured with 10% of rice bran wax loaded with bamboo fiber of 40 µm (EVO-BF_40_), extra-virgin olive oil structured with 10% of rice bran wax loaded with bamboo fiber of 90 µm (EVO-BF_90_), and extra-virgin olive oil structured with 10% of rice bran wax loaded with bamboo fiber of 150 µm (EVO-BF_150_). Different letters next to the bars indicate significant differences among mean values (*p* < 0.05).

**Figure 4:**
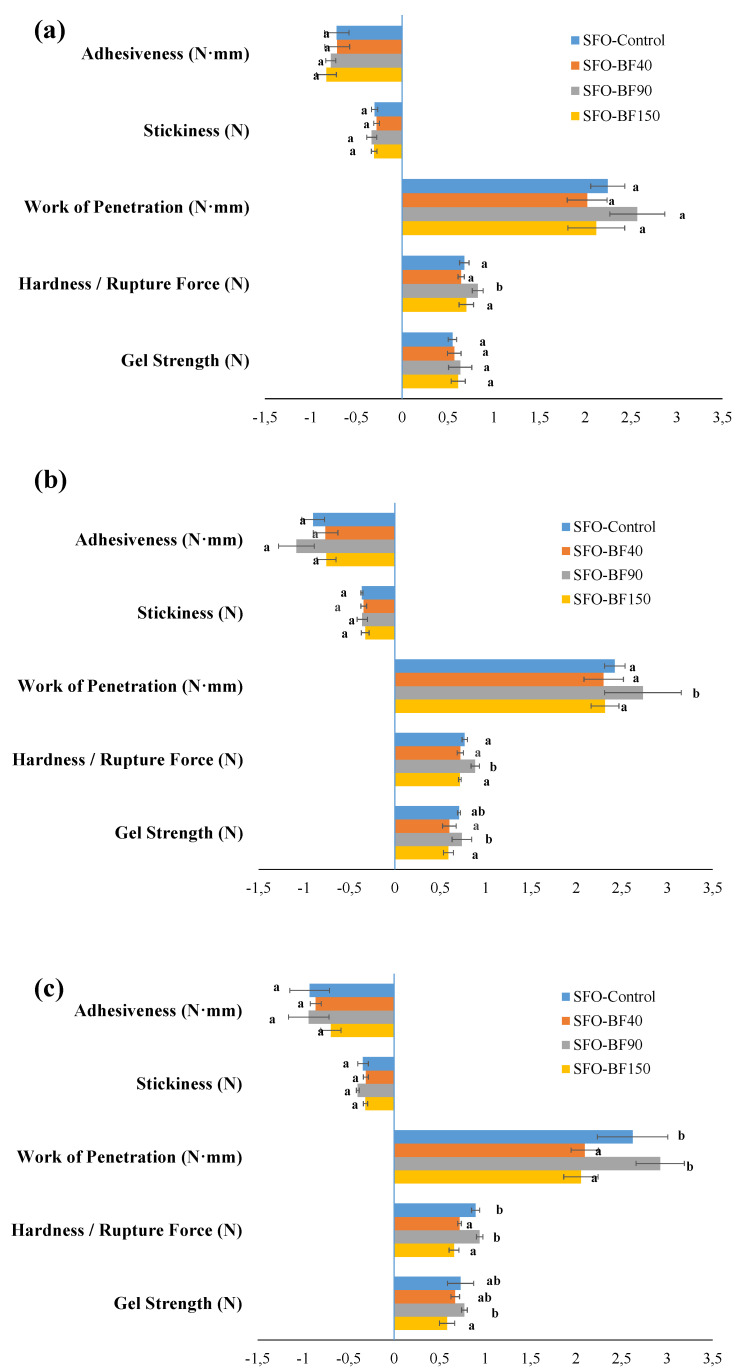
Values of gel strength (N), hardness, (N), work of penetration (N∙mm), stickiness (N), and adhesiveness (N∙mm), for the sunflower oil-based gels prepared at 25 °C (**a**), 4 °C (**b**), and 0 °C (**c**), as a function of the BF average dimension. The following systems were investigated: sunflower oil structured with 10% of rice bran wax (SFO-Control), sunflower oil structured with 10% of rice bran wax loaded with bamboo fiber of 40 µm (SFO-BF_40_), sunflower oil structured with 10% of rice bran wax loaded with bamboo fiber of 90 µm (SFO-BF_90_), and sunflower oil structured with 10% of rice bran wax loaded with bamboo fiber of150 µm (SFO-BF_150_). Different letters next to the bars indicate significant differences among mean values (*p* < 0.05).

The mechanical properties of gelled materials were assessed at low deformation (up to 50%) according to a prior study by Pang et al. [8] in order to correctly mediate the effect of both external and internal surface structure on texture. In particular, values of textural attributes as a function of either fiber addition or cooling temperature were reported for EVO- (Figure 3) and SFO- (Figure 4) based systems, respectively. Interestingly, for the sake of literature comparison, the order of magnitude of both gel strength and hardness for EVO-based gels was consistently lower than those characterizing systems prepared with other structuring agents, such as carnauba wax [32] and soybean wax esters [33], whereas a good agreement with monoglycerides-structured samples was found [32]. However, fiber addition of either low (BF_40_) or intermediate (BF_90_) granulometry determined a significant (*p* < 0.05) increase in texture attributes over only RBW-based gels at both 25 °C and 4 °C, thus suggesting the occurrence of structural reinforcement of the network, even though no statistical differences (*p* > 0.05) could be retrieved in terms of stickiness and adhesiveness values (Figure 3). A possible explanation for the achieved results can arise from considering the effect of both PSD and the ratio between the soluble and insoluble fiber content of added BF. Specifically, in their work on the application of high-pressure homogenization (HPH) on the microstructural and techno-functional properties of orange pulp and peel fiber powders, Huang et al. [34] demonstrated that the structural comminution induced by HPH processing led to a reduction in bulk density with a consequent promotion of water/oil holding capabilities, which then improved emulsifying ability via steric effects and/or electrostatic repulsion mechanism. Instead, in a more recent study, David et al. [35] used different botanical cellulose fibers to texturize rapeseed oil without employing any thermal treatment. The authors demonstrated that 30% *w*/*w* dispersions of bamboo and wheat cellulose powders granted a structuring effect to the oil and, hence, created stable systems without any oil clearance. In addition, microscopic analyses performed on the achieved gels revealed that fiber with a predominant insoluble component was able to create a sharp front between the oil phase and concentrated solid-like dispersion, giving evidence of the oleo-gelation mechanism.

When the gelation of EVO-based systems was induced by cooling at 0 °C, the structuring effect associated with fiber addition was completely leveled off, regardless of the considered BF granulometry. It is also worth highlighting the influence of cooling temperature on the network structure of achieved oleogels. To this end, in all cases decreasing the cooling temperature down to 0 °C ensured a drop in gel stability and, consequently, lower firmness. Macroscale textural properties better highlighted the strong dependency of network strength on temperature conditions. In particular, lowering the cooling temperature has a significant detrimental effect on gel tridimensional structure that causes the reduction in texture attributes. The latter result was in disagreement with the outcomes belonging to the study of Blake and Marangoni [10], where rice bran wax (RBX)-, sunflower wax (SFX)-, and candelilla wax (CLX)-based oleogels were cooled down at two different rates, namely 5 °C/min and 1 °C/min, respectively. The authors noticed that faster cooling promoted smaller crystals formation with a highly tortuous network that positively affected structure and oil-binding capacity. In our case, while texture reduction was prominent for control and BF_150_ gels, systems prepared with BF_40_ and BF_90_ maintained the structure unchanged up to 4 °C, thus corroborating the use of fibers not only as structuring agents but also as a stabilizer against temperature fluctuation.

As far as SFO-based systems are concerned, the fiber effect on their mechanical properties was evident only when cooling was performed at 4 °C and 0 °C (Figure 4). Specifically, at 25 °C similar results in terms of investigated texture parameters than those detected for control samples were recorded after BF_90_ addition within the starting formulation, with the presence of BF_40_ and BF_150_ denoting a weakening in gel network strength (*p* < 0.05). Plus, BF_90_-based gels exhibited a significant soaring (*p* > 0.05) in both hardness and work of penetration attributes when cooling temperature decreased down to the minimum adopted one. Nevertheless, the temperature influence on structural parameters reported in Figure 4 had less impact than that observed in Figure 3, thus suggesting a different crystallization mechanism between sunflower and olive oil (Figure 3 and Figure 4). The latter results found coherence with what was already reported in other studies as a function of wax melting properties [36,37], oil viscosity, and oil/wax affinity [38].

### 3.3. DSC Analysis

DSC analysis was carried out to unravel the effect of fiber presence on the thermal properties of gelled systems. In full agreement with previous literature studies [15,36,39], all thermograms reported two main peaks, which are an endothermic one present in the heating ramp for a temperature range of 58–70 °C, and an exothermic peak throughout the cooling step at around 60–64 °C, which shows the occurrence of rice bran wax melting/crystallization phenomena. However, the oil type was the only variable affecting the peak temperatures. More specifically, the melting peak showed a maximum at around 66.6 °C for EVO gels and 67.5 °C for SFO gels, while the peak temperatures during crystallization were found to be equal to 63.2 °C and 61.8 °C, respectively. Data extrapolated from DSC thermograms and associated with enthalpies of melting (ΔH*_m,heating_*) and crystallization (ΔH*_c,cooling_*) were reported for EVO- and SFO-based systems in Table 2 and Table 3. Results showed that the oleogels were not ideally thermo-reversible during repeated heating and cooling cycles, as differences in the averaged values of either melting or crystallization temperature/enthalpy were observed.

From Table 2, it is clear that fiber inclusion at any granulometry within the oil phase, at both 25 °C and 4 °C of cooling temperature, involved a significant (*p* < 0.05) increase in melting enthalpies, which seems to suggest a greater network resistance, thus reinforcing the previously shown results of Figure 3. The relationship between melting enthalpies and firmness was recently reported in previous studies. For instance, Yang et al. [40] claimed that the increase in the melting enthalpy of oleogel samples as a function of the gelator mixture (β-sitosterol and stearic acid) concentration was associated with results of the microscopic structure and firmness. Similar results in terms of a direct correlation between texture firmness and melting enthalpies were also found by Valoppi et al. [41] while studying the effect of 10% (*w*/*w*) monoglycerides addition in castor, cod liver, corn, and extra-virgin olive oils on the formation and structural features of organogels.

Additionally, in our case, except for EVO-based control gel, the reported values of melting/crystallization enthalpies decreased by lowering the cooling temperature. This corroborated the negative effect of temperature reported also in texture measurement (Figure 3). Overall, minor differences as a function of the fiber content, as well as cooling temperature, were detected for sunflower gel systems (Table 3).

### 3.4. Rotational Rheological Tests

**Figure 5:**
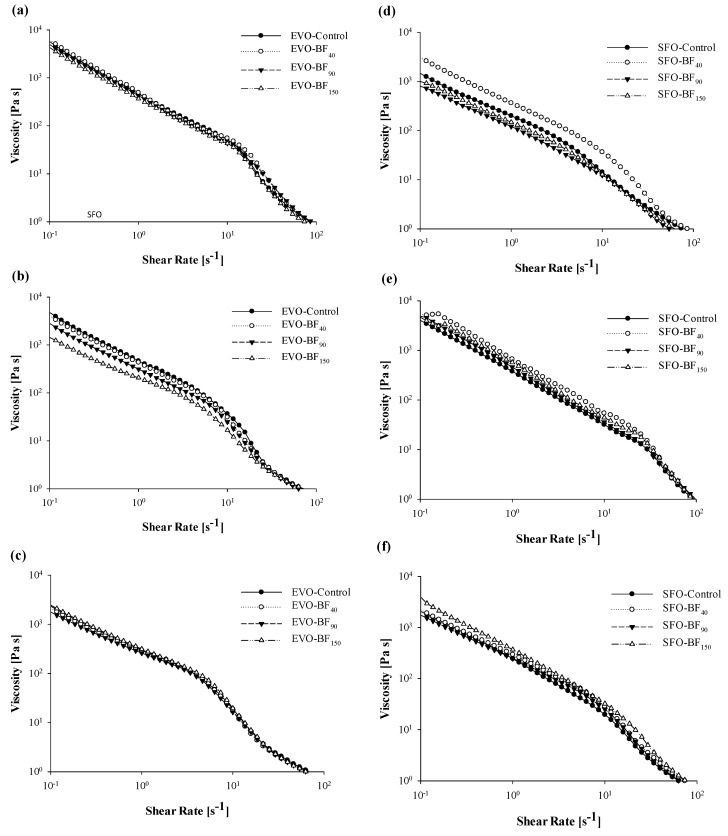
Curves of viscosity (*η*, Pa·s) against the shear rate ($\dot{\gamma }$,s^−1^) for the extra-virgin olive oil- (**a**–**c**) and sunflower oil- (**d**–**f**) based gels as a function of cooling temperature: 25 °C (**a**,**d**), 4 °C (**b**,**e**) and 0 °C (**c**,**f**). Rheological measurements were performed at 25 °C. The following systems were investigated: Extra-virgin olive oil structured with 10% of rice bran wax (EVO-Control), extra-virgin olive oil structured with 10% of rice bran wax loaded with bamboo fiber of 40 µm (EVO-BF_40_), extra-virgin olive oil structured with 10% of rice bran wax loaded with bamboo fiber of 90 µm (EVO-BF_90_), and extra-virgin olive oil structured with 10% of rice bran wax loaded with bamboo fiber of 150 µm (EVO-BF_150_); sunflower oil structured with 10% of rice bran wax (SFO-Control), sunflower oil structured with 10% of rice bran wax loaded with bamboo fiber of 40 µm (SFO-BF_40_), sunflower oil structured with 10% of rice bran wax loaded with bamboo fiber of 90 µm (SFO-BF_90_), and sunflower oil structured with 10% of rice bran wax loaded with bamboo fiber of 150 µm (SFO-BF_150_).

Oleo-gelation substantially modified the rheological behavior of oil samples, being the predominant component within product formulation. In particular, all the investigated samples exhibited a pseudo-plastic behavior, characterized by a decreasing trend of viscosity as a function of the shear rate (Figure 5), which departs from the typical Newtonian behavior shown by EVO and SFO oils with viscosity barely dependent on the shear rate [42,43]. The shear-thinning behavior in wax-based oleogel was previously detected by Doan et al. [39] when exploiting different wax sources (including RBW) to be incorporated inside the oil system, as well as by Gur et al. [44] and Ögütcü et al. [45,46] for sunflower oil/rice bran wax and olive oil/beeswax gels, respectively. Moreover, it is known that the pseudo-plastic rheological model is applicable for other oleogelated systems based on structuring agents such as ethyl-cellulose [47] and β-sitosterol [48]. However, some discrepancies among flow curves of samples were underlined, especially at the highest investigated cooling temperatures, since an almost complete overlapping is noticed when either dropping the set cooling condition to 0 °C or shifting the shear rate towards values greater than 10 s^−1^ (Figure 5).

In order to properly describe the rheological behavior of gels via useful parameters, viscosity vs. shear rate curves were analyzed using Ostwald power-law rheological models [49], as coherently reported in Equation (3).

(3) $$\eta =K{\dot{\gamma }}^{n-1}\;\;$$

where *η* is the apparent viscosity [Pa ∙ s], $\dot{\gamma }$ is the shear rate [s^−1^], *K* is the so-called consistency index [Pa ∙ s*^n^*], and *n* is the dimensionless flow index.

As can be seen from the results schematized in Table 4 and Table 5, significant (*p* < 0.05) differences among samples can be detected in terms of both the flow index (*n*) and consistency index (*K*) values, regardless of the investigated edible oil system. More in detail, for EVO gels cooled down at room temperature, it was remarkable to notice how *K* values associated with systems added by BF_90_ and BF_150_ exhibited the highest standard deviation, thus suggesting a greater structural instability and, hence, hypothesizing the theory according to which longer fibers negatively interact during network consolidation. Discrepancies among samples were more evident when performing cooling at 4 °C, since BF_90_ and BF_150_ consistency index stood 1.6-fold to 2-fold below that of control samples, respectively. Furthermore, the effect of temperature was visible particularly for control and BF40 with a significant decrease (*p* < 0.05) in viscosity, while BF_90_ and BF_150_ consistency were constant along with different solidification conditions (Table 4). Instead, for SFO gels, at both 25 °C and 4 °C of cooling temperature, BF_40_-added systems exhibited the highest values of *K* (*p* < 0.05) as compared to other samples, while differences were completely lost at 0 °C (Table 5).

**Figure 6:**
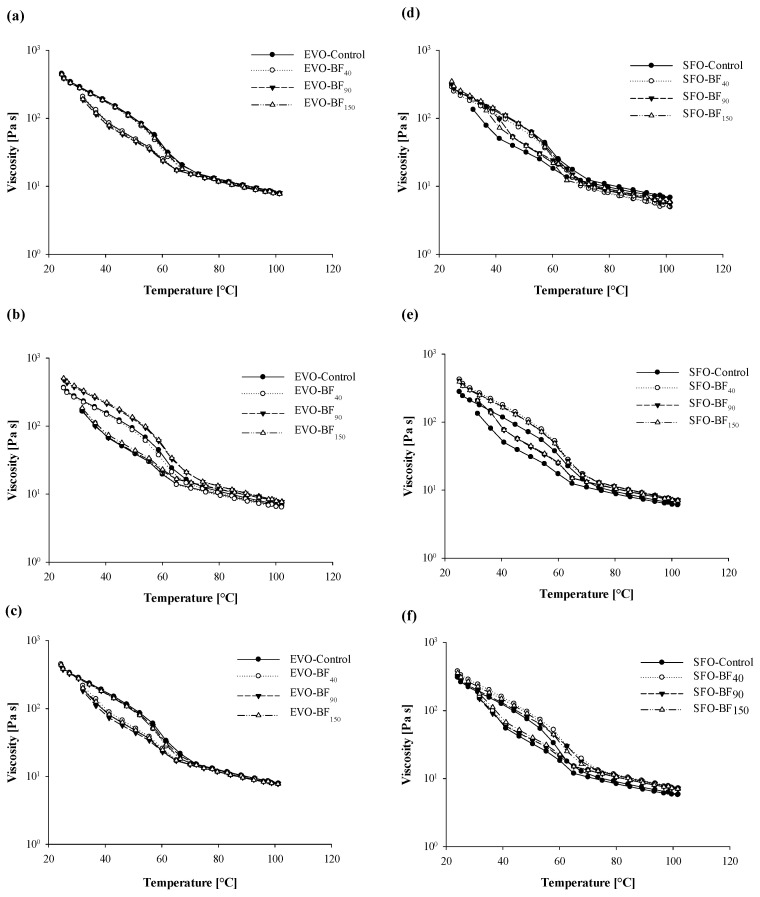
Curves of viscosity (*η*, Pa·s) against the temperature (°C) for the extra-virgin olive oil-(**a**–**c**) and sunflower oil- (**d**–**f**) based gels as a function of cooling temperature: 25 °C (**a**,**d**), 4 °C (**b**,**e**), and 0 °C (**c**,**f**). Rheological measurements were performed at a constant shear rate of 50 s^−1^. The following systems were investigated: extra-virgin olive oil structured with 10% of rice bran wax (EVO-Control), extra-virgin olive oil structured with 10% of rice bran wax loaded with bamboo fiber of 40 µm (EVO-BF_40_), extra-virgin olive oil structured with 10% of rice bran wax loaded with bamboo fiber of 90 µm (EVO-BF_90_) and extra-virgin olive oil structured with 10% of rice bran wax loaded with bamboo fiber of 150 µm (EVO-BF_150_), sunflower oil structured with 10% of rice bran wax (SFO-Control), sunflower oil structured with 10% of rice bran wax loaded with bamboo fiber of 40 µm (SFO-BF_40_), sunflower oil structured with 10% of rice bran wax loaded with bamboo fiber of 90 µm (SFO-BF_90_), and sunflower oil structured with 10% of rice bran wax loaded with bamboo fiber of 150 µm (SFO-BF_150_).

With the aim to better understand the viscous properties of the oleogel samples during the cooling process, viscosity ramps as a function of temperature were performed and results are depicted in Figure 6. For the edible oil whose viscosity followed an Arrhenius-like behavior [50], oleogels showed a change in the slope above 60 °C in agreement with DSC melting results (Table 2 and Table 3).

Similar outcomes were achieved by Sahu et al. [51] in a study on the utilization of unsaponifiable matter obtained from rice bran oil for preparing an antioxidant-rich oleogel. Likewise, Saha et al. [50], comparing the rheological properties of the oil, oil, and wax suspension with wax oleogel and oleo-foam, noticed that for temperatures above 70 °C the viscosity of all the investigated systems was similar, thus confirming that most solid content has melted. Conversely for lower temperatures, the viscosity of the oleogel and oleo-foam deviate significantly from both oil and wax suspension.

The viscosity assumed a closed-loop pattern during thermal treatment, indicating the thermo-reversibility of systems [17,45]. Nevertheless, all samples exhibited a drop in viscosity during the cooling ramp indicating the presence of hysteresis and suggesting structural modification during heating/cooling cycles as reported in Figure 6. For EVO-based gels, no differences were detected at 25 °C and 0 °C, while at 4 °C BF_90_ and BF_150_ exhibited wider hysteresis compared to control and BF_40_ (Figure 6a–c), which corroborates the findings from rotational rheology reported in Table 4. Conversely, SFO hysteresis was more dependent on cooling temperature. Specifically, at 25 °C all fiber-added samples showed a narrower area between heating and cooling ramp compared to control (Figure 6d), while at 4 °C and 0 °C no differences were detected (Figure 6e,f).

### 3.5. Oscillatory Rheological Tests

**Figure 7:**
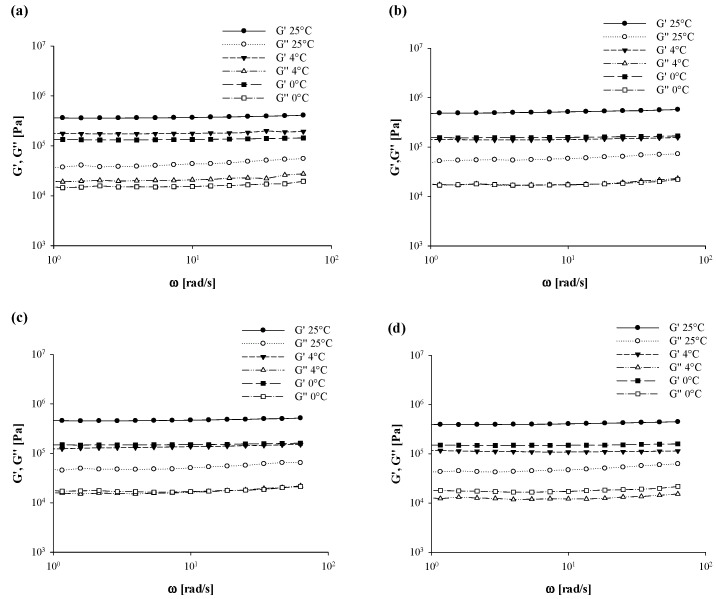
Curves of elastic modulus (*G*′, Pa) and loss modulus (*G*″, Pa) against the frequency (*ω*, rad/min) for the extra-virgin oil-based gels as a function of the average dimension of added fiber and adopted cooling temperature. Legend: Extra-virgin oil structured with 10% of rice bran wax (EVO-Control) (**a**), extra-virgin oil structured with 10% of rice bran wax loaded with bamboo fiber of 40 µm (EVO-BF_40_) (**b**), extra-virgin oil structured with 10% of rice bran wax loaded with bamboo fiber of 90 µm (EVO-BF_90_) (**c**), extra-virgin oil structured with 10% of rice bran wax loaded with bamboo fiber of 150 µm (EVO-BF_150_) (**d**).

**Figure 8:**
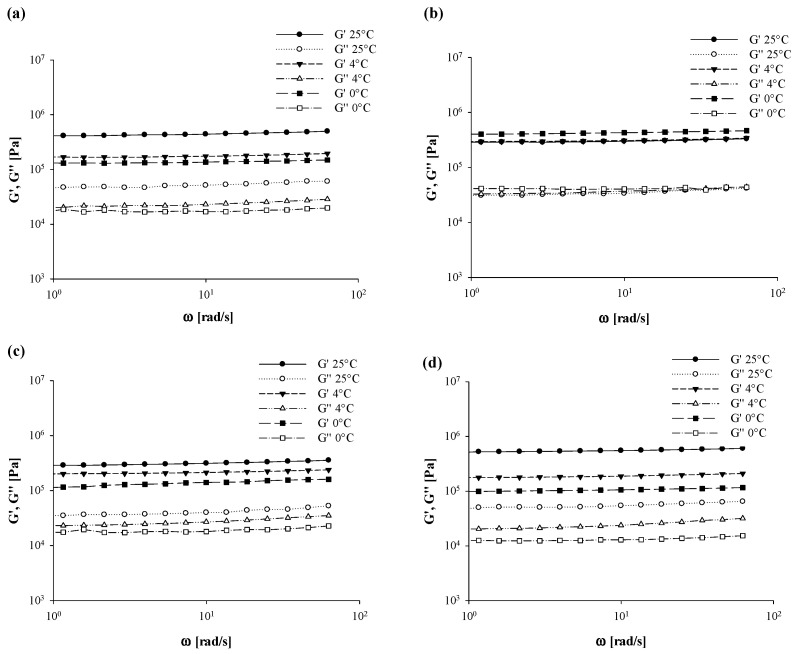
Curves of elastic modulus (*G*′, Pa) and loss modulus (*G*″, Pa) against the frequency (*ω*, rad/min) for the sunflower oil-based gels as a function of the average dimension of added fiber and adopted cooling temperature. Legend: sunflower oil structured with 10% of rice bran wax (SFO-Control) (**a**), sunflower oil structured with 10% of rice bran wax loaded with bamboo fiber of 40 µm (SFO-BF_40_) (**b**), sunflower oil structured with 10% of rice bran wax loaded with bamboo fiber of 90 µm (SFO-BF_90_) (**c**), sunflower oil structured with 10% of rice bran wax loaded with bamboo fiber of 150 µm (SFO-BF_150_) (**d**).

The averaged values of oscillatory rheological parameters (*G*′ and *G*″) of the achieved oleogels as a function of either the cooling temperature or the granulometry of added BF are reported in Figure 7 (EVO-based systems) and Figure 8 (SFO-based systems).

Regardless of the investigated samples, a solid-like behavior with storage modulus higher than loss modulus (*G*′ > *G*″) at any cooling condition was found, thus suggesting that a more elastic structure was formed. According to Tavernier et al. [52], systems being characterized by a ratio between *G*″ and *G*′ lower than 0.1 could be defined as a “strong gel”. Moreover, both moduli exhibited a slight dependency from the frequency, with no crossover point detection (Figure 7 and Figure 8).

The latter results seem to confirm the stability of gels due to the cross-linking of the three-dimensional network as a formation mechanism of the oleogel, as previously stated by Doan et al. [36]. Similar findings were illustrated by Xia et al. [53] when testing binary systems of carnauba wax (CW) and 1-docosanol (DS) at different ratios for oleogels production. The authors observed a stronger interaction between CW and DS under the ratio of 1:4, which then helped to form a more compact three-dimensional network, thus resulting in greater oscillatory properties. On the same line, in a study on the evaluation of the physical properties of different types of wax/oil systems as a function of the oil (olive, corn, soybean, sunflower, safflower, and canola), wax (sunflower oil wax (SFOW), paraffin wax (PW), and beeswax (BW) at different concentration (1% to 10%) types, Martini et al. [54] found that sunflower wax (SFOW) samples resulted in higher values of *G*′ as a consequence of the higher amount of crystalline material generated during the crystallization phase.

Data from Figure 7 and Figure 8 also highlight the effect of cooling temperature on structural parameters of both EVO- and SFO-based gels, since a significant (*p* < 0.05) reduction in both *G*′ and *G*″ values took place when moving from 25 °C to 0 °C. However, in spite of comparable EVO gels with and without fiber addition (Figure 7), a more evident effect of cooling temperature when varying BF granulometry was disclosed in correspondence with SFO-based systems.

In this research work, the manipulation of *G*′ and *G*″ vs. frequency data enabled us to introduce further rheological parameters, as reported by Equation (4) using gel-system models as reported by Gabriele et al. [55], in order to reinforce the findings of Figure 7 and Figure 8:

(4) G*(ω)=G′(ω)2+G″(ω)2=AF·ω1z 

More specifically, G* is the complex modulus [Pa], *G*′ and *G*″ are the elastic and loss modulus [Pa], respectively, *ω* is the frequency [rad/s], *A_F_* stands for the gel strength [Pa∙s^1/*z*^], and 1/*z* is the inverse of coordination degree which gives a clear idea of network extension.

Data obtained from weak-model regression were reported in Table 6 and Table 7, respectively, for EVO- and SFO-based systems. When considering the former samples, it can be seen that the 1/*z* was not significantly affected by BF addition (*p* < 0.05) up to 25 °C, while some discrepancies among samples were detected at lower cooling temperatures, which then suggests dissimilar network extensions. In particular, as reported by Gabriele [55], a lower value of 1/*z* indicated a greater number of interactions. In this specific case, BF_40_ and BF_90_ exhibited the lowest values of 1/*z*, thus indicating the greatest 3-D extensions at 4 °C, with similar results being found for BF_150_ at 0 °C (Table 6).

Major dissimilarities were noticed in terms of the *A_F_* parameter, especially at 25 °C of cooling temperature, whose intensity increased on average by 30% due to BF_40_ and BF_90_ addition as compared to control samples, hence corroborating the hypothesis of an increase in gel strength as confirmed by texture (Figure 3) and DSC (Table 2) analyses. On the other hand, such accordance among oscillatory results, differential calorimetry, and texture analysis was not kept unaltered for SFO-based systems. As a matter of fact, employing BF_40_ and BF_150_ within oleogel formulation yielded the lowest values of 1/*z* at 25 °C whereas, in contrast with the outcome of texture analysis (Figure 4), different (*p* < 0.05) A_F_ values over control samples following BF addition were observed. However, by decreasing cooling temperature, BF_40_-based systems uniquely maintained their properties almost intact, and a significantly (*p* < 0.05) consistent drop in gel strength was detected for all the other samples (Table 7). According to Co and Marangoni [17], the type of oil strongly affects the gelation properties since an efficient gelator needs to balance its solubility and insolubility in a solvent to achieve proper gelator–gelator and gelator–oil interactions. A common example is given by the poor compatibility between RBW and rice bran oil found by Doan et al. [39] and recently confirmed by Tavernier et al. [37] and Wijarnprecha et al. [14]. However, it is useful to remember that oscillatory tests are mostly used to measure the stability of food systems over time, rather than solely depicting a fixed time situation. Nonetheless, the typical frequency range between 0.1 Hz and 100 Hz is very useful because it corresponds roughly to the time scale of industrial interest [55,56]. Thus, the discrepancies of sunflower oil systems might derive from different time-scale behavior.

**Figure 9:**
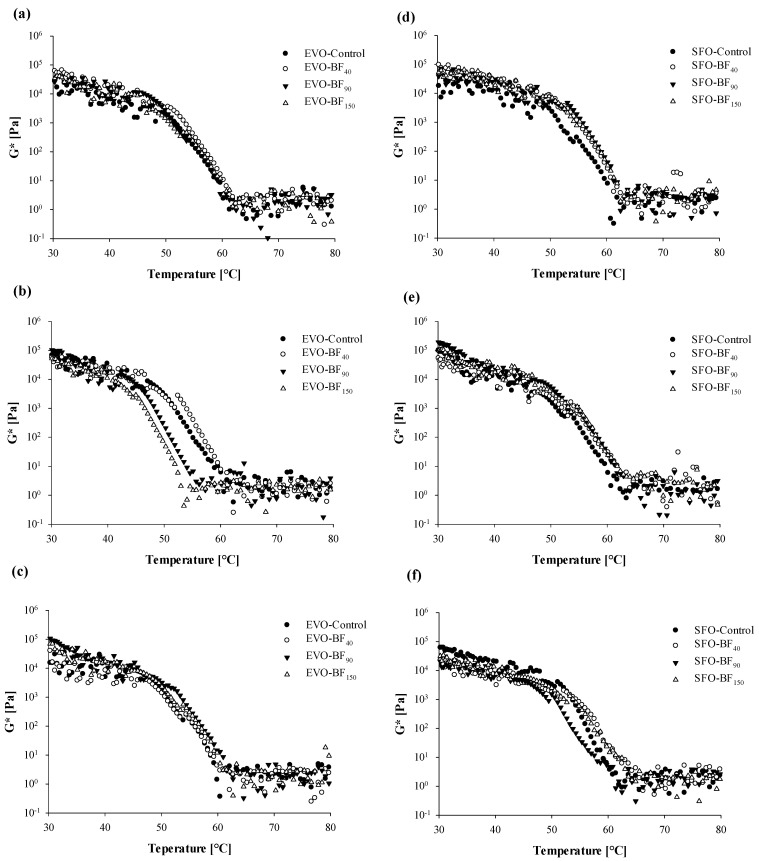
Curves of complex modulus (G*, Pa) against the temperature (°C) for the extra-virgin olive oil- (**a***–***c**) and sunflower- (**d**–**f**) based gels as a function of the adopted cooling temperature: 25°C (**a**,**d**), 4 °C (**b**,**e**), and 0 °C (**c**,**f**). The following systems were investigated: extra-virgin olive oil structured with 10% of rice bran wax (EVO-Control), extra-virgin olive oil structured with 10% of rice bran wax loaded with bamboo fiber of 40 µm (EVO-BF_40_), extra-virgin olive oil structured with 10% of rice bran wax loaded with bamboo fiber of 90 µm (EVO-BF_90_), and extra-virgin olive oil structured with 10% of rice bran wax loaded with bamboo fiber of 150 µm (EVO-BF_150_); sunflower oil structured with 10% of rice bran wax (SFO-Control), sunflower oil structured with 10% of rice bran wax loaded with bamboo fiber of 40 µm (SFO-BF_40_), sunflower oil structured with 10% of rice bran wax loaded with bamboo fiber of 90 µm (SFO-BF_90_), and sunflower oil structured with 10% of rice bran wax loaded with bamboo fiber of 150 µm (SFO-BF_150_).

In order to gain more insights into the stability of gel samples, curves of complex modulus (G*) as a function of temperature were achieved and reported in Figure 9. These data refer to information needed to observe changes in the storage and loss moduli during the sample heating process under constant strain and frequencies, thus evidencing how the oleogels respond to temperature variation. Irrespective of the considered system, the G* values underwent a smooth linear decrease as the temperature was raised to 50 °C. However, inside the temperature span of 30–50 °C, the samples were well solidified and gelled. As soon as the temperature was further increased (T > 50 °C), gels started to soften and complete melting occurred after 60 °C, as seen in the abrupt drop in G* curves encountered throughout Figure 9. Instead, going beyond 60 °C, the oleogels became free-flowing liquids with the G* values remaining almost constant.

A similar trend was highlighted from the previous research on wax-based oleogels. In particular, Barroso et al. [57] found the same melting behavior in a study on a combination of berry (BEW) or sunflower wax (SHW) with glycerol monostearate (GMS) in flaxseed oil (FXO). Plus, Yilmaz et al. [58], investigating the rheological performance of animal wax-based oleogels, reported the same change in structure along with temperature increase. In disagreement with the findings of Yilmaz and Demirci [59], temperature sweep data did not seem to concur with DSC-determined melting peak points (around 66–67 °C), but more with crystallization temperature (61–63 °C). Moreover, additional discrepancies are also highlighted at fixed cooling temperature conditions when adopting BF (Figure 9). DSC data provide only melting onset and peak temperatures and enthalpies, thus rheological temperature ramp graphics allow for following the evolution of gel structure during the heating process. In their study on animal wax-based oleogels, Yilmaz et al. [58] noticed that DSC melting temperature (Tm) values for the oleogel samples were much lower than ones reported by the temperature sweep test in the occurrence of the crossover point. The authors hypothesized that, even after melting, some gelled consistency might remain, associated with junction zones of the waxes to detectable yields. Additionally, in their work on the characterization of wax and hydrolyzed wax-based oleogels, Wettlaufer et al. [60] reported that the temperatures derived from under shear (oscillation) rheology were lower than those detected via the quiescent DSC-based data, possibly relating to the fact that initial crystallization is not necessarily equivalent to the appearance of a space filling network.

Our data also show the different thermal stability of the investigated oleogel systems. For instance, in the case of EVO-based samples, a neat division between two groups in terms of achieved trends was detected. In particular, the addition of BF_90_ and BF_150_ anticipated the occurrence of softening and melting phenomena in comparison with either control or BF_40_-based systems (Figure 9b). These outcomes are well correlated with viscosity vs. temperature data (Figure 6b) for which wider hysteresis was associated with greater microstructural instability. To further confirm the latter results in samples cooled at 25 °C and 0 °C for which any difference in hysteresis area was detected, no variation in terms of temperature sweep behavior was highlighted (Figure 9a,c). Conversely, SFO hysteresis was more dependent on cooling temperature. Specifically, at 25 °C all fiber-added samples showed a narrower area between the heating and cooling ramp compared to control (Figure 5d), while at 4 °C and 0 °C no differences were detected (Figure 5e,f). Similarly, SFO curves at 25 °C also showed a neat difference between control and fiber-added gels, with a visible shift towards higher temperatures, as reported by temperature sweep data (Figure 9d). Finally, further differences were detected at very low cooling temperatures (0 °C), and the stability of the gel system progressively followed the given order of BF_150_ < Control < BF_90_ < BF_40_.

## 4. Conclusions

In general, lowering the cooling temperature had a significant detrimental effect on gel three-dimensional structure which caused a loss in terms of texture and rheology, as demonstrated from both rotational and oscillatory mode tests. Nevertheless, fibers, especially at low granulometry, played a crucial role as both structuring agents and as a stabilizer against temperature fluctuation. Specifically, in olive oil gel systems, BF**_40_** and BF**_90_** exhibited a significant increase in texture properties that was linked to an increase in terms of melting energy detected by DSC analysis and resulted in greater gel strength values. In the case of the sunflower oil system, even if a hardening effect was detected by BF**_90_** addition, no similar correlations were detected, suggesting a different affinity between oil/wax systems as well as different kinetics in crystallization due to gelator–gelator and gelator–oil interactions.

From a rheological point of view, all samples showed a pseudo-plastic behavior regardless of the investigated cooling temperature and fiber addition. Hence, all gels exhibited a thermo-reversible structure with the presence of hysteresis, suggesting a partial loss in gel structure during the heating/cooling structure.

Further investigation is needed to track the evolution of the crystallization mechanism and the influence of solid fat content on the achieved microstructure, as well as to better elucidate the role of fiber presence and cooling temperature in network formation. Moreover, additional tests are strictly required with the aim to optimize product formulation by reducing wax concentration.

## Figures and Tables

**Table 1:** Values of water holding capacity (WHC), sunflower (SOHC) and olive oil (OOHC) holding capacity, and D_4,3_ of commercial bamboo fibers utilized throughout this study.

	WHC [g/g]	SOHC [g/g]	OOHC [g/g]	D_4,3_ [µm]
BF_40_	2.96 ± 0.15 ^a^	2.84 ± 0.05 ^a^	3.54 ± 0.15 ^a^	38.70 ± 0.02 ^a^
BF_90_	3.27 ± 0.35 ^a^	3.86 ± 0.02 ^c^	4.10 ± 0.07 ^b^	64.96 ± 1.53 ^b^
BF_150_	3.23 ± 0.26 ^a^	3.64 ± 0.05 ^b^	4.44 ± 0.30 ^b^	61.82 ± 1.59 ^b^

Bamboo fiber of 40 µm (BF_40_), bamboo fiber of 90 µm (BF_90_), and bamboo fiber of 150 µm (BF_150_). For each investigated parameter, values reported within the same column with different lowercase letters are significantly different (*p* < 0.05).

**Table 2:** Values of melting (ΔH*_m,heating_*) and crystallization (ΔH*_c,cooling_*) enthalpies of extra-virgin olive oil gelled systems, obtained by either heating or cooling cycles, arising from DSC thermograms elaboration.

	ΔH_*m heating*_ [J/g]			ΔH_*c,cooling*_ [J/g]		
	25 °C	4 °C	0 °C	25 °C	4 °C	0 °C
EVO-Control	15.26 ± 0.06 ^aA^	16.23 ± 0.34 ^aB^	16.60 ± 0.18 ^aB^	8.46 ± 0.26 ^abA^	8.05 ± 0.01 ^aA^	7.78 ± 0.29 ^aA^
EVO-BF_40_	18.20 ± 0.09 ^cB^	18.11 ± 0.09 ^bB^	16.11 ± 0.42 ^aA^	8.72 ± 0.07 ^bB^	8.57 ± 0.20 ^bAB^	8.09 ± 0.08 ^aA^
EVO-BF_90_	18.23 ± 0.03 ^cB^	17.92 ± 0.20 ^bB^	15.68 ± 0.15 ^aA^	7.77 ± 0.16 ^aA^	8.17 ± 0.03 ^abA^	8.10 ± 0.12 ^aA^
EVO-BF_150_	17.84 ± 0.00 ^bB^	17.92 ± 0.22 ^bB^	16.30 ± 0.17 ^aA^	7.90 ± 0.27 ^abA^	8.26 ± 0.02 ^abAB^	8.86 ± 0.01 ^bB^

The following systems were investigated: extra-virgin olive oil structured with 10% of rice bran wax (EVO-Control), extra-virgin olive oil structured with 10% of rice bran wax loaded with bamboo fiber of 40 µm (EVO-BF_40_), extra-virgin olive oil structured with 10% of rice bran wax loaded with bamboo fiber of 90 µm (EVO-BF_90_), and extra-virgin olive oil structured with 10% of rice bran wax loaded with bamboo fiber of 150 µm (EVO-BF_150_). For each investigated parameter, values with different lowercase letters within the same column (effect of fiber addition) and values with different uppercase letters within the same row (effect of cooling temperature) are significantly different (*p* < 0.05).

**Table 3:** Values of melting (ΔH*_m,heating_*) and crystallization (ΔH*_c,cooling_*) enthalpies of sunflower gelled systems obtained by either heating or cooling cycles, arising from DSC thermograms elaboration.

	ΔH_*m,heating*_ [J/g]			ΔH_*c,cooling*_ [J/g]		
	25 °C	4 °C	0 °C	25 °C	4 °C	0 °C
SFO-Control	15.49 ± 0.33 ^aA^	15.92 ± 0.11 ^aA^	15.50 ± 0.52 ^bA^	7.97 ± 0.28 ^aA^	7.90 ± 0.03 ^aA^	8.11 ± 0.26 ^aA^
SFO-BF_40_	15.62 ± 0.13 ^aA^	16.06 ± 0.59 ^aA^	16.18 ± 0.01 ^bA^	8.33 ± 0.01 ^abA^	8.60 ± 0.01 ^abA^	8.04 ± 0.60 ^aA^
SFO-BF_90_	15.65 ± 0.24 ^aA^	15.83 ± 0.14 ^aA^	15.41 ± 0.84 ^bA^	8.51 ± 0.18 ^abB^	8.72 ± 0.26 ^bB^	7.38 ± 0.08 ^aA^
SFO-BF_150_	15.67 ± 0.09 ^aB^	15.91 ± 0.05 ^aB^	12.39 ± 0.01 ^aA^	8.86 ± 0.02 ^bB^	8.72 ± 0.27 ^bAB^	8.11 ± 0.01 ^aA^

The following systems were investigated: sunflower oil structured with 10% of rice bran wax (SFO-Control), sunflower oil structured with 10% of rice bran wax loaded with bamboo fiber of 40 µm (SFO-BF40), sunflower oil structured with 10% of rice bran wax loaded with bamboo fiber of 90 µm (SFO-BF90), and sunflower oil structured with 10% of rice bran wax loaded with bamboo fiber of 150 µm (SFO-BF150). For each investigated parameter, values with different lowercase letters within the same column (effect of fibre addition) and values with different uppercase letters within the same row (effect of cooling temperature) are significantly different (*p* < 0.05).

**Table 4:** Values of flow index (*n*) and consistency index (*K*) of extra-virgin olive oil gelled systems, obtained from the flow curves data regression by the power-law model (Equation (3)).

	*_n_* [-]			*K* [Pa∙s^n^]		
	25 °C	4 °C	0 °C	25 °C	4 °C	0 °C
EVO-Control	0.24 ± 0.02 ^aAB^	0.29 ± 0.01 ^bB^	0.22 ± 0.02 ^aA^	449 ± 7 ^aB^	400 ± 4 ^cB^	243 ± 30 ^aA^
EVO-BF_40_	0.24 ± 0.01 ^aA^	0.29 ± 0.03 ^bA^	0.23 ± 0.02 ^aA^	477 ± 7 ^aC^	355 ± 29 ^bcB^	241 ± 7 ^aA^
EVO-BF_90_	0.17 ± 0.06 ^aA^	0.23 ± 0.03 ^abA^	0.19 ± 0.02 ^aA^	341 ± 118 ^aA^	260 ± 38 ^abA^	207 ± 2 ^aA^
EVO-BF_150_	0.21 ± 0.09 ^aA^	0.15 ± 0.07 ^aA^	0.24 ± 0.04 ^aA^	353 ± 120 ^aA^	188 ± 54 ^aA^	255 ± 38 ^aA^

Rheological measurements were performed at 25 °C. The following systems were investigated: extra-virgin olive oil structured with 10% of rice bran wax (EVO-Control), extra-virgin olive oil structured with 10% of rice bran wax loaded with bamboo fiber at 40 µm (EVO-BF_40_), extra-virgin olive oil structured with 10% of rice bran wax loaded with bamboo fiber at 90 µm (EVO-BF_90_), and extra-virgin olive oil structured with 10% of rice bran wax loaded with bamboo fiber at 150 µm (EVO-BF_150_). For each investigated parameter, values with different lowercase letters within the same column (effect of fiber addition) and values with different uppercase letters within the same row (effect of cooling temperature) are significantly different (*p* < 0.05).

**Table 5:** Values of flow index (*n*) and consistency index (*K*) of sunflower oil gelled systems, obtained from the flow curves data regression by the power-law model (Equation (3)).

	*_n_* [-]			*K* [Pa∙s^n^]		
	25 °C	4 °C	0 °C	25 °C	4 °C	0 °C
SFO-Control	0.15 ± 0.01 ^aAB^	0.20 ± 0.01 ^bB^	0.11 ± 0.04 ^aA^	361 ± 62 ^aB^	211 ± 30 ^bA^	164 ± 28 ^aA^
SFO-BF_40_	0.21 ± 0.01 ^bB^	0.17 ± 0.02 ^bAB^	0.17 ± 0.01 ^aA^	600 ± 82 ^bB^	325 ± 42 ^cA^	250 ± 17 ^aA^
SFO-BF_90_	0.18 ± 0.02 ^abB^	0.06 ± 0.01 ^aA^	0.15 ± 0.03 ^aB^	436 ± 56 ^aC^	104 ± 6 ^aA^	211 ± 14 ^aB^
SFO-BF_150_	0.17 ± 0.02 ^bB^	0.09 ± 0.04 ^aA^	0.20 ± 0.04 ^aB^	496 ± 75 ^abC^	126 ± 13 ^aA^	316 ± 114 ^aB^

Rheological measurements were performed at 25 °C. The following systems were investigated: sunflower oil structured with 10% of rice bran wax (SFO-Control), sunflower oil structured with 10% of rice bran wax loaded with bamboo fiber of 40 µm (SFO-BF40), sunflower oil structured with 10% of rice bran wax loaded with bamboo fiber of 90 µm (SFO-BF90), and sunflower oil structured with 10% of rice bran wax loaded with bamboo fiber of 150 µm (SFO-BF150). For each investigated parameter, values with different lowercase letters within the same column (effect of fiber addition) and values with different uppercase letters within the same row (effect of cooling temperature) are significantly different (*p* < 0.05).

**Table 6:** Values of network strength (*A*) and network extension (1/*z*) of extra-virgin olive oil gelled systems, obtained from the frequency sweep curves data regression by the weak-gel model (Equation (4)).

	*A_F_*·10^−5^ [Pa]			1/*z*·10^2^ [-]		
	25 °C	4 °C	0 °C	25 °C	4 °C	0 °C
EVO-Control	3.56 ± 0.76 ^aC^	2.06 ± 0.24 ^abB^	1.32 ± 0.14 ^aA^	2.0 ± 0.2 ^aA^	3.0 ± 0.4 ^bB^	2.0 ± 0.2 ^abA^
EVO-BF_40_	4.81 ± 0.37 ^bB^	1.41 ± 0.50 ^abA^	1.54 ± 0.10 ^aA^	4.0 ± 0.8 ^aB^	2.0 ± 0.2 ^aA^	2.0 ± 0.3 ^abAB^
EVO-BF_90_	4.51 ± 0.79 ^bB^	2.54 ± 0.48 ^bA^	1.47 ± 0.30 ^aA^	3.0 ± 0.5 ^aB^	2.0 ± 0.2 ^aA^	2.0 ± 0.1 ^bA^
EVO-BF_150_	3.50 ± 0.22 ^aB^	1.16 ± 0.23 ^aA^	1.49 ± 0.33 ^aA^	3.0 ± 0.2 ^aB^	2.0 ± 0.4 ^abAB^	1.0 ± 0.1 ^aA^

Rheological measurements were performed at 25 °C. The following systems were investigated: Extra-virgin oil structured with 10% of rice bran wax (EVO-Control), extra-virgin oil structured with 10% of rice bran wax loaded with bamboo fiber at 40 µm (EVO-BF_40_), extra-virgin oil structured with 10% of rice bran wax loaded with bamboo fiber at 90 µm (EVO-BF_90_), and extra-virgin oil structured with 10% of rice bran wax loaded with bamboo fiber at 150 µm (EVO-BF_150_). For each investigated parameter, values with different lowercase letters within the same column (effect of fiber addition) and values with different uppercase letters within the same row (effect of cooling temperature) are significantly different (*p* < 0.05).

**Table 7:** Values of network strength (*A*) and network extension (1/*z*) of sunflower oil gelled systems, obtained from the frequency sweep curves data regression by the weak-gel model (Equation (4)).

	*A_F_*·10^−5^ [Pa]			1/*z*·10^2^ [-]		
	25 °C	4 °C	0 °C	25 °C	4 °C	0 °C
SFO-Control	4.11 ± 0.33 ^bcB^	1.49 ± 0.58 ^aA^	1.31 ± 0.43 ^aA^	4.0 ± 0.3 ^bB^	4.0 ± 0.2 ^aB^	3.0 ± 0.2 ^aA^
SFO-BF_40_	3.39 ± 0.28 ^abA^	2.46 ± 0.79 ^aA^	4.00 ± 0.40 ^bA^	3.0 ± 0.2 ^aA^	3.0 ± 0.3 ^aA^	3.0 ± 0.1 ^aA^
SFO-BF_90_	2.85 ± 0.54 ^aB^	1.97 ± 0.19 ^aAB^	1.15 ± 0.30 ^aA^	5.0 ± 0.3 ^bA^	4.0 ± 0.4 ^aA^	10.0 ± 1.5 ^bB^
SFO-BF_150_	5.22 ± 0.39 ^cC^	1.76 ± 0.65 ^aB^	0.77 ± 0.22 ^aA^	3.0 ± 0.3 ^aA^	4.0 ± 0.3 ^aA^	5.0 ± 1.4 ^aA^

Rheological measurements were performed at 25 °C. The following systems were investigated: Sunflower oil structured with 10% of rice bran wax (SFO-Control), sunflower oil structured with 10% of rice bran wax loaded with bamboo fiber of 40 µm (SFO-BF40), sunflower oil structured with 10% of rice bran wax loaded with bamboo fiber of 90 µm (SFO-BF90), and sunflower oil structured with 10% of rice bran wax loaded with bamboo fiber of 150 µm (SFO-BF150). For each investigated parameter, values with different lowercase letters within the same column (effect of fiber addition) and values with different uppercase letters within the same row (effect of cooling temperature) are significantly different (*p* < 0.05).
